# Mesoscale modelling of miscible and immiscible multicomponent fluids

**DOI:** 10.1038/s41598-019-44745-8

**Published:** 2019-06-04

**Authors:** Z. C. Zhao, R. J. Moat, R. S. Qin

**Affiliations:** School of Engineering & Innovation, The Open University, Walton Hall, Milton Keynes, MK7 6AA UK

**Keywords:** Chemical engineering, Coarse-grained models

## Abstract

A mesoscopic simulation method based on the integration of dissipative particle dynamics (DPD), smoothed particle hydrodynamics (SPH) and computational thermodynamics (CT) has been developed. The kinetic behaviours of miscible and immiscible fluids were investigated. The interaction force between multicomponent mesoscopic particles is derived from the system free energy. The diffusivity of the components in non-ideal solution is determined by the chemical potential. The proposed method provides convincing predictions to the effects of convection, diffusion and microscopic interaction on the non-equilibrium evolution of engineering fluids, and demonstrates a potential to simulate more complicated phenomena in materials processing.

## Introduction

Multicomponent fluids contain miscible and/or immiscible elements. The mixing and demixing phenomena are important in chemical engineering and material fabrication^1,2^. Many advanced materials are fabricated by precise control of kinetic evolution. For example, a core-shell nanostructured metallic glass with supreme plasticity was fabricated by well-controlled demixing processing^3^, while an aluminium-bismuth alloy with uniformly distributed Bi particle relies on well-controlled mixing procedure to improve its bearing ability^4^. On the contrary, a poorly controlled process degrades the materials properties^5^.

The kinetic evolution in mixing and demixing is affected by convection, diffusion and thermodynamics of the materials. The diffusion-convection equation illustrates the coherent interaction among the factors^6^

1 $$\frac{\partial c}{\partial t}=\nabla \cdot (D\nabla c)-\nabla (\overrightarrow{v}c)+{R}_{s}$$

where *D* is the diffusivity, $\overrightarrow{v}$ the convection velocity and *R*_*s*_ is the sources or sinks of the composition. *R*_*s*_ is dependent on the reactive thermodynamics. Materials microscopic interaction affects the viscosity and diffusivity^7,8^. Convection influences distribution of compositions, which subsequently affects the phase transformation. The redistribution of composition affects the force field between compositions and hence to affect flow pattern. The kinetic evolution of multicomponent material should be described by an integrated model compromising at least the diffusion, convection and thermodynamics.

Many numerical methods have been developed to tackle this problem under various approximations. Phase field model has been implemented to study the mixing and demixing processes either without convection^9^ or with a largely simplified hydrodynamic description of flow behaviour^10^. Finite element method has been integrated with phase-field model to study the micro-segregation in the solidification of binary alloys^11^. The approach faces difficult in description of realistic cases such as that in blast furnace, where the flow is laminar at one location and tubular in another with some complex geometries of the phase boundaries. Lattice Boltzmann equation has demonstrated the capability in description of the mixing and demixing process^12,13^, which is applicable to multiphase and multicomponent systems^14,15^ but facing challenges in dealing with non-isothermal, high density-ratio and high Reynolds number systems. The dissipative particle dynamics (DPD) and smooth particle hydrodynamics (SPH) are off-lattice hydrodynamic methods^16,17^ capable of simulating multiphase fluid with no need of explicit interface tracking^18,19^. However, their capability to the incorporation of engineering thermodynamics needs to be developed further. There is no generic method reported in literature to allow the integration of the mesoscopic simulation method to commercial thermodynamic database that has been implemented widely in engineering.

This work aims to develop an integrated method based on the frameworks of DPD, SPH and computational thermodynamics, and use the method to investigate the effect of convection, diffusion and thermodynamics on the materials kinetic behaviour in mixing and demixing processes. The purpose of the research was to simulate materials processing in engineering cases.

## Results

The material is represented approximately by a pack of mesoscopic interactive particles. Each particle contains many atoms and is a subsystem that obeys the statistical thermodynamics. Inheriting the basic idea from DPD method, the interaction between a pair of mesoscopic particles contains the conservative, dissipative and random forces^20^

2 $${\overrightarrow{F}}_{ij}={\overrightarrow{F}}_{ij}^{c}+{\overrightarrow{F}}_{ij}^{D}+{\overrightarrow{F}}_{ij}^{R}$$

where the sub-index represents the particle *i* and *j* respectively. The dissipative force *F*^*D*^ represents the viscosity. The random force *F*^*R*^ represents the thermal fluctuation. The fluctuation-dissipation theorem can be satisfied if *F*^*D*^ and *F*^*R*^ are correlated adequately^20^. The kinetic behaviour of an ideal solution can be reproduced by Eq. (2) using ${\overrightarrow{F}}_{ij}^{c}=0$. $\,{F}_{ij}^{c}$ represents the interaction between particles. Given the fact that many engineering materials are processed in an open environment under approximately constant pressure, the state thermodynamic quantity in such an environment is Gibbs free energy. The equivalent mesoscopic driving force in kinetic evolution can be derived from the thermodynamic quantity as^21^

3 $${\overrightarrow{F}}_{ij}=-\,\frac{\delta {G}_{ij}}{\delta {r}_{ij}}{\hat{r}}_{ij}$$

where *G*_*ij*_ = *G*_*j*_−*G*_*i*_ is the Gibbs free energy difference between two points in the space. Equation (3) means that the mesoscopic driving force ${\overrightarrow{F}}_{ij}$ intends to drive the particle to move between two different points. This can be considered as two nearby mesoscopic particles *i* and *j*. ${\hat{r}}_{ij}=({\overrightarrow{r}}_{j}-{\overrightarrow{r}}_{i})/|{\overrightarrow{r}}_{j}-{\overrightarrow{r}}_{i}|$, where ${\overrightarrow{r}}_{i}$ is the spatial vector at the mass centre of particle *i*. $G=\int g(c,\,T)dv$, where *g*(*c*, *T*) is the Gibbs free energy density and *c* = {*c*_1_, *c*_2_, … *c*_*n*_} for the material containing *n* components. For a material without ferromagnetic element one has^22^

4 $$g(c,T)={g}^{ideal}(c,T)+{g}^{ex}(c,T)={g}^{bulk}(c,T)+{g}^{id}(c,T)+{g}^{ex}(c,T)$$

where *g*^*ideal*^(*c*, *T*) = *g*^*bulk*^(*c*, *T*) + *g*^*id*^(*c*, *T*) is the free energy density of ideal material, ${g}^{bulk}(c,T)=\sum _{\alpha =1}^{n}\,{c}_{\alpha }{g}_{\alpha }(T)$ is the free energy of bulk pure elements, ${g}^{id}(c,T)=RT\sum _{\alpha =1}^{n}\,{c}_{\alpha }ln{c}_{\alpha }$ is the free energy due to ideal mixing and *g*^*ex*^(*c*, *T*) is called the excess free energy density. R is the gas constant. *g*_*α*_(*T*) is the free energy density of pure element α at temperature *T*, which is available for all the elements^22^. Substituting Eq. (4) into (3) and comparing with (2), one has

5 $${\overrightarrow{F}}_{ij}^{C}=-\,\frac{\delta {G}_{ij}^{ex}}{\delta {r}_{ij}}{\hat{r}}_{ij}$$

where ${G}^{ex}=\int {g}^{ex}(c,\,T)dv$. Equation (5) has the same format as that of derived by Pagonabarraga and Frenkel^23^. In the computational thermodynamics, the excess free energy density is expressed as Redlich-Kister expansion^24^

6 $${g}^{ex}(c,T)=RT\,\sum _{k=1}^{n-1}\,\sum _{l=k+1}^{n}\,[{c}_{k}{c}_{l}\sum _{m=0}^{p\,}\,{L}_{m}^{kl}{({c}_{k}-{c}_{l})}^{m}]$$

where ${L}_{m}^{kl}$ is the m-order interactive coefficient between element *k* and *l* and is dependent on the temperature. The value of ${L}_{m}^{kl}$ are available for many materials in commercial thermodynamic databases^25^. For a binary fluid, Eq. (6) reduces into ${g}^{ex}({\rm{c}},{\rm{T}})={\rm{RT}}\,{{\rm{c}}}_{{\rm{A}}}{{\rm{c}}}_{{\rm{B}}}\sum _{{\rm{m}}=0}^{{\rm{p}}}\,{{\rm{L}}}_{{\rm{m}}}^{{\rm{AB}}}{({{\rm{c}}}_{{\rm{A}}}-{{\rm{c}}}_{{\rm{B}}})}^{{\rm{m}}}$. For binary regular solution, it further reduces into Porter equation ${G}^{ex}=RT{{\rm{L}}}_{0}^{{\rm{AB}}}{{\rm{c}}}_{{\rm{A}}}{{\rm{c}}}_{{\rm{B}}}$. The convection of an engineering fluid can be calculated according to the derived mesoscopic interactive force.

The governing equation for solute diffusion was derived by Cahn as $\frac{dc}{dt}=\nabla [M\nabla (\frac{\partial g}{\partial c})]=\nabla [M\frac{{\partial }^{2}g}{\partial {c}^{2}}\nabla (c)]$ ^26^, where *M* is a positive coefficient called mobility and the diffusivity is defined as $D=M\frac{{\partial }^{2}g}{\partial {c}^{2}}$ in comparison with Fick’s law. *D* ≥ 0 when $\frac{{\partial }^{2}g}{\partial {c}^{2}}\ge 0$, which corresponds to solute mixing in miscible fluid. *D* < 0 when $\frac{{\partial }^{2}g}{\partial {c}^{2}} < 0$, which corresponds to the case of spinodal decomposition. The more generic case was derived from the irreversible law of thermodynamics where the effect of inter-diffusion on the volume change was taken into account^7^. In the present approximation, materials are represented by moving mesoscopic particles. The chemical composition and temperature in different mesoscopic particles might be different to reflect the heterogeneous non-equilibrium materials. The free energy density and hence the diffusivity in each mesoscopic particle can be in different values. The diffusion between two adjacent particles *i* and *j* requires to determine its diffusivity *D*_*ij*_ according to the respective diffusivity *D*_*i*_ and *D*_*j*_. To this purpose one considers a fictitious mid-point position *r*_*m*_ between *i* and *j* positions, the diffusivity from *r*_*i*_ to *r*_*m*_ is *D*_*i*_ and from *r*_*j*_ to *r*_*m*_ is *D*_*j*_. The net solute flux from both *i* and *j* particles to *r*_*m*_ should be zero according to mass conservative, i.e.

7 $${D}_{i}\frac{({c}_{m}-{c}_{i})}{\,|{r}_{m}-{r}_{i}|}+{D}_{j}\frac{({c}_{m}-{c}_{j})}{\,|{r}_{m}-{r}_{j}|}=0$$

Equation (7) gives ${c}_{m}=\frac{{D}_{i}{c}_{i}+{D}_{j}{c}_{j}}{{D}_{i}+{D}_{j}}$. The flux from *i* to *j* is ${D}_{j}\frac{({c}_{j}-{c}_{m})}{\,|{r}_{j}-{r}_{m}|}+{D}_{i}\frac{({c}_{m}-{c}_{i})}{\,|{r}_{m}-{r}_{i}|}=\frac{2{D}_{j}{D}_{i}}{{D}_{i}+{D}_{j}}\frac{({c}_{j}-{c}_{i})}{|{r}_{j}-{r}_{i}|}$. This gives

8 $${D}_{ij}=\frac{2{D}_{i}{D}_{j}}{{D}_{i}+{D}_{j}}$$

The diffusivity can be in positive or negative values that are dependent on the chemical constitution, composition and temperature. Therefore, it is possible that *D*_*i*_ + *D*_*j*_ = 0 in some cases. Equation (8) goes infinite when *D*_*i*_ = −*D*_*j*_. In such case, The mass conservation in Eq. (7) cannot be satisfied if *c*_*i*_ ≠ *c*_*j*_ with *D*_*i*_ ≠ 0 and *D*_*j*_ ≠ 0. To guarantee the mass conservation, it requires *D*_*i*_ = 0 and *D*_*j*_ = 0. This will enable Eq. (8) to be not infinite because *D*_*i*_*D*_*j*_ is a higher order infinite small quantity than *D*_*i*_ + *D*_*j*_. In summary, the diffusivity between adjacent particles *i* and *j* is

9 $${D}_{ij}=\{\begin{array}{ll}\frac{2{D}_{i}{D}_{j}}{{D}_{i}+{D}_{j}} & \,when\,{D}_{i}+{D}_{j}\ne 0\\ 0 & \,when\,{D}_{i}+{D}_{j}=0\end{array}$$

With Eq. (9) in mind and also to represent the diffusion equation into following format

10 $$\frac{dc}{dt}=\frac{1}{\rho }\nabla (D\rho \nabla {\rm{c}})$$

Numerical solution of Eq. (10) can be achieved using SPH methodology. The latter uses interpolation method and introduces a kernel to calculate the quantity and the associate derivatives. Equation (10) in SPH is represented as^26^

11 $$\frac{d{c}_{i}}{dt}=\sum _{j}\,\frac{2{D}_{ij}{m}_{j}}{{\rho }_{i}{\rho }_{j}}\frac{({\rho }_{i}+{\rho }_{j})({c}_{i}-{c}_{j})}{{\overrightarrow{r}}_{ij}}\nabla W({r}_{ij},h)\,$$

where *m*_*i*_ and *ρ*_*i*_ are the mass and density of particle *i*. *h* is the smooth length and *W* is the kernel function. There are several possible formats of *W* available to choose from^17^.

In the numerical calculation, one chooses ${\overrightarrow{F}}_{ij}^{D}=-\,\gamma {\omega }^{D}({r}_{ij})({\overrightarrow{r}}_{ij}\cdot {\overrightarrow{v}}_{ij}){\overrightarrow{r}}_{ij}$ and ${\overrightarrow{F}}_{ij}^{R}=\sigma {\omega }^{R}({r}_{ij})\zeta {\overrightarrow{r}}_{ij}$ ^20^, where ${\overrightarrow{v}}_{ij}={\overrightarrow{v}}_{j}-{\overrightarrow{v}}_{i}$, ${\overrightarrow{e}}_{ij}={\overrightarrow{r}}_{ij}/|{\overrightarrow{r}}_{ij}|$. *ζ* is the white noise between −0.5 and 0.5 and with 0 mean. *γ* and *σ* are coefficients satisfying *σ*^2^ = 2*γk*_*B*_*T*, *k*_*B*_ and T are the Boltzmann constant and temperature, respectively. *ω*^*D*^ and *ω*^*R*^ are weight functions satisfying

12 $${\omega }^{R}({r}_{ij})=\sqrt{{\omega }^{D}({r}_{ij})}=\{\begin{array}{ll}1-\frac{{r}_{ij}}{h} & \,({r}_{ij}\le h)\\ 0 & \,({r}_{ij} > h)\end{array}$$

13 $$W({r}_{ij},h)=\{\begin{array}{ll}\frac{1}{4\pi {h}^{3}}[{(2-{r}_{ij}/h)}^{3}-4{(1-{r}_{ij}/h)}^{3}] & (0\le {r}_{ij}\le h)\\ \frac{1}{4\pi {h}^{3}}{(2-{r}_{ij}/h)}^{3} & \,(h < {r}_{ij}\le 2h)\\ 0\, & (2h < {r}_{ij})\end{array}$$

The theoretical derivations presented in the earlier paragraphs have demonstrated that the method proposed in the present work is based on an integration of DPD, SPH and CT. The kinetic evolution of the system was described following a DPD strategy, where conservative, dissipative and random forces are implemented to describe the driving force. The relationship between dissipative force and random force are defined according to classical DPD theory. However, the chemical constitution and its evolution are described according to the SPH framework, where interpolation method and kernel function are implemented. The conservative force and the diffusivity are derived and obtained according to the CT theory and the associate format of database. The method developed in the present work is based on integration of DPD, SPH and CT.

If the chemical diffusion is neglected by letting *D*_*i*_ = 0 and conservative force is defined as ${\overrightarrow{F}}_{ij}^{c}={a}_{ij}{\omega }^{R}({r}_{ij})$ with *a*_*ij*_ a constant and *ω*^*R*^(*r*_*ij*_) the same format as that defined in Eq. (12), the newly developed method in the present work reduces to the classical DPD method that was discussed by Groot and Warren^20^. Their results can be reproduced if the same coefficients are chosen because the governing equations for both models in such conditions are identical^20^. The numerical results for particle demixing at various ratios of particle numbers have been demonstrated in Supplementary Figures. On the other side when the coefficients of dissipative and random forces are defined as *γ* = 0 and *σ* = 0, the newly developed method will reduce to conventional SPH where kernel interpolation is applied to calculate all the quantities (e.g. mass of particle) and their spatial derivations (e.g. gradient of particle density). The thermodynamic quantity represented in Eq. (6) is widely applied in description of condensed matters such as solid and liquid metallic materials. For gaseous phases, activities and partial pressure can be used. All of those are well described in computational thermodynamics.

To implement the integrated model developed in the present work, one considers a regular binary solution with solute A and solvent B. For a mesoscopic particle containing *N*_*i*_ atoms of regular binary elements with solute mass composition *c*_*i*_, the excess free energy is $${G}_{i}^{ex}={N}_{i}{K}_{B}T{{\rm{L}}}_{0}^{{\rm{AB}}}{c}_{i}(1-{c}_{i})$$ and diffusivity is $$D=M\frac{{\partial }^{2}{g}_{i}}{\partial {c}_{i}^{2}}=M{K}_{B}T$$$$(\frac{1}{{c}_{i}}+\frac{1}{1-{c}_{i}}-2{{\rm{L}}}_{0}^{{\rm{AB}}})$$. Its mass is obtained by $${m}_{i}={N}_{i}[{c}_{i}\cdot {m}_{A}+(1-{c}_{i})\cdot {m}_{B}]=({N}_{i}/{N}_{A})$$$$[{c}_{i}\cdot {m}_{A}+(1-{c}_{i})\cdot {m}_{B}]$$, where *m*_*A*_ and *m*_*B*_ are the atom mass of element A and B respectively, *N*_*A*_ is the Avogadro’s constant. Its density, without considering of the mixture-induced volume change, can be obtained by $${\rho }_{i}={c}_{i}\cdot {\rho }_{A}+(1-{c}_{i})\cdot {\rho }_{B}$$, where *ρ*_*A*_and *ρ*_*B*_ are mass density of pure element A and B respectively.

For total *N*_*t*_ number of mesoscopic particles in a cubic box, one defines the dimensionless units for the convenience of numerical calculation. The dimensionless mass reduces into ${m}_{i}^{\ast }={m}_{i}/\{{N}_{t}[c\cdot {m}_{A}+(1-c)\cdot {m}_{B}]\}$, where *c* is the average composition. The temperature reduces into ${T}_{i}^{\ast }={T}_{i}^{\ast }/{T}_{m}$ where *T*_*m*_ is the melting temperature of a binary mixture with the average composition. The free energy density is reduced to ${g}_{i}^{\ast }={g}_{i}/(R{T}_{m})$. The smooth length reduces to h* = *h*/*h*_*c*_, where ${h}_{c}=\sqrt[3]{{N}_{t}[c{m}_{A}+(1-c){m}_{A}]/\{{L}_{x}{L}_{y}{L}_{z}[c{\rho }_{A}+(1-c){\rho }_{A}]\}}$ and *L*_*x*_, *L*_*y*_ and *L*_*z*_ are number of unit cells in each coordinates. Based on the above definition, one has t* = *t*/*τ*, where $\tau =\sqrt{{m}_{p}\cdot {r}_{c}^{2}/({K}_{B}{T}_{m})}$. It gets ${D}_{i}^{\ast }={D}_{i}\tau /{h}_{c}^{2}$ and ${M}^{\ast }={D}_{i}^{\ast }/({K}_{B}{T}_{m})$.

**Figure 1:**
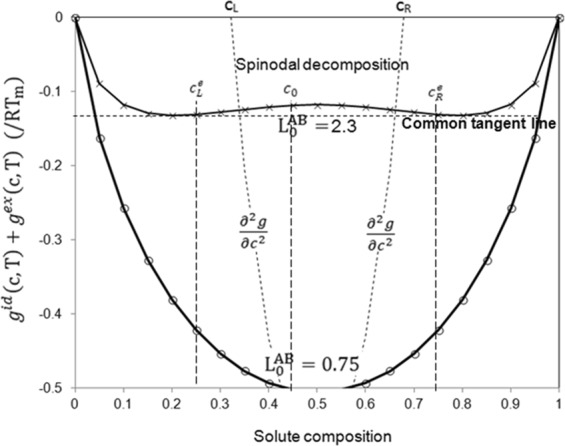
The change of g^id^(c, T) + g^ex^(c, T) as a function of solute composition shows that ${{\rm{L}}}_{0}^{{\rm{AB}}}=0.75$ is miscible and ${{\rm{L}}}_{0}^{{\rm{AB}}}=2.3$ is immiscible. ∂^2^*g*/∂*c*^2^ = 0 reveals the spinodal decomposition area between *c*_*L*_ and *c*_*R*_. For the immiscible case, the cross points between common tangent line and Gibbs free energy curve gives the equilibrium compositions ${c}_{L}^{e}$ and ${c}_{R}^{e}$. The cross point between the comment tangent line and the average chemical composition determines the minimum Gibbs free energy.

For the parameters of *N*_*t*_ = 4000, *L*_*x*_ = *L*_*y*_ = *L*_*z*_ = 5 and *c* = 0.45, *N*_*i*_ = 10 gives average mass of mesoscopic particle of *m*_*i*_ = 1.01 × 10^−24^ kg and *h* = 15.35 Å for Cu-Ni liquid miscible alloy and *m*_*i*_ = 1.945 × 10^−24^ kg and *h* = 21.06 Å for Al-Pb liquid immiscible alloy. Cu-Ni alloy is well-known for its unlimited miscibility of one element in another in liquid or solid states. The alloy has been developed for many decades and used as coinage, electrical components and other occasions^27^. Al-Pb is an immiscible binary alloy^28^. The mechanical mixture of Al-Pb is desirable for making wear parts in automotive industry. Both alloys have been studied intensively by experiments. The melting temperature for Cu-Ni is 1573 K and for Al-Pb about 1643 K at the given solute composition of *c* = 0.45. One chooses *σ* = 3 and *γ* = *σ*^2^/2 for random force and dissipative force according to the recommendation in literature^20^, and selects ${{\rm{L}}}_{0}^{{\rm{AB}}}=0.75$ for miscible alloy and ${{\rm{L}}}_{0}^{{\rm{AB}}}=2.3$ for immiscible alloy. The selection of ${{\rm{L}}}_{0}^{{\rm{AB}}}$ in the present simulation didn’t follow the actual value of specific materials for the purpose to prove general applicability of the proposed method. This is because ${{\rm{L}}}_{0}^{{\rm{AB}}}$ is not only materials-related but also temperature-dependent. More importantly, several interactive coefficients are required to determine the non-regular and non-ideal materials. However, the values are in the range of real conditions. The computational thermodynamic methodology is followed strictly in the following studies. The calculation of phase diagram (CALPHAD) based on computational thermodynamics has been proved to agree with experimental characterization of phase diagram in many engineering cases. Giving the fact that the bulk free energy doesn’t usually affect the phase equilibrium, one plotted *g*^*id*^(*c*, *T*) + *g*^*ex*^(*c*, *T*) in Fig. 1. It shows clearly that ${{\rm{L}}}_{0}^{{\rm{AB}}}=0.75$ corresponds to a miscible fluid at any solute composition but ${{\rm{L}}}_{0}^{{\rm{AB}}}=2.3$ represents an immiscible fluid. Spinodal decomposition happens when the average solute composition is between *c*_*L*_ and *c*_*R*_. To selection of diffusivity is after the reference of true values of realistic system, e.g. the diffusivity of dilute Cu-Ni alloy of 3.27 × 10^−9^ m^2^s^−1^ and that of dilute Al-Pb of 1.0 × 10^−6^ m^2^s^−1^ respectively^9,29^, one has M* = 0.0144 and M* = 5.84 for the two systems. Periodical boundary condition was implemented in the simulations.

**Figure 2:**
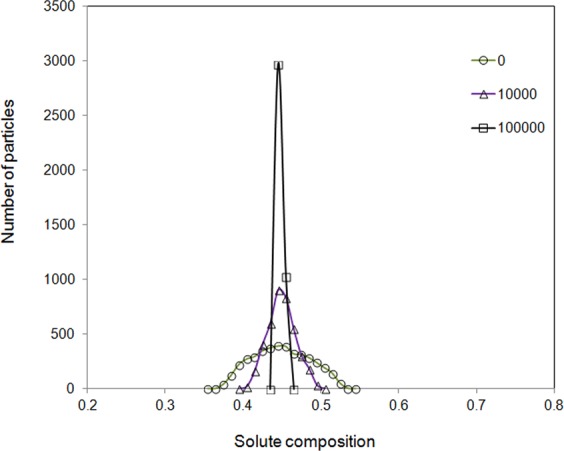
Distribution of particle numbers with different solute compositions at 0, 1 × 10^4^ and 1 × 10^5^ time steps for miscible fluid.

The histogram of particle concentration distribution for miscible system is demonstrated in Fig. 2. At the beginning, an average solute composition of 0.45 with a range of variation between 0.4 and 0.5 was assigned to the mesoscopic particles randomly. The solute composition axis from 0 to 1 was divided into 100 parts and the mesoscopic particles were counted within each solute part by a statistical code program. For example, those particles with solute composition between 0.4 and 0.41 were considered as the same solute composition of 0.405 during the plotting of Fig. 2. As the time evolves, more and more particles have their solute composition to approach to the average value. The variation range decreases and the distribution peak becomes sharp, which indicates a gradual mixing of solute in the solvent. After 10^5^ time steps, a single peak in the histogram was nearly formed. There are 2970 particles with composition between 0.44 and 0.45, and 1030 particles with composition between 0.45 and 0.46. This suggests that the mixing was almost reached and solute were distributed almost homogeneously.

**Figure 3:**
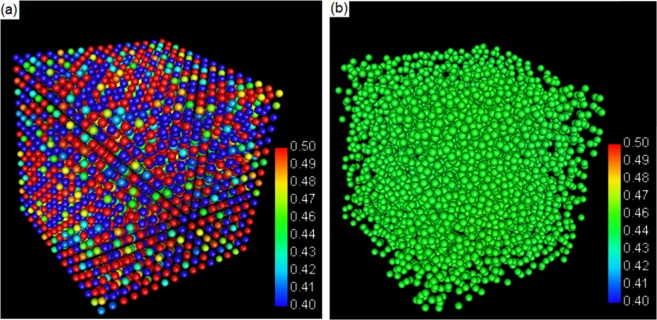
Configurations of mesoscopic particles at (**a**) 0 and (**b**) 1 × 10^5^ time steps. The colour spectrum corresponds to the solute concentration from 0.4 to 0.5.

The 3D configurations of particles at 0 and 1 × 10^4^ time steps were plotted respectively using MatVisual, as are presented in Fig. 3(a,b). The colour of the particles is determined by the solute composition in a range between 0.4 and 0.5, with 0.4 in blue and 0.5 in red at the colour spectrum. The colours were distributed randomly in Fig. 3(a), which demonstrates the random initialization of the solute composition to mesoscopic particles. The colours are converged toward that in the middle of spectrum after 1 × 10^4^ time steps, as shown in Fig. 3(b). The other time steps demonstrated in Fig. 2 were also plotted but not presented here because the Fig. 3(b) has already shows the tendency of solute homogenization clearly.

**Figure 4:**
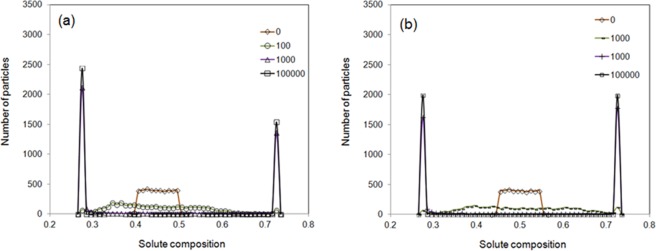
Distribution of particle numbers with various solute compositions at 0, 100, 1000 and 1 × 10^5^ time steps for immiscible fluids. (**a**) The average solute composition is 0.45. (**b**) The average solute composition is 0.5.

The histogram for the distribution of particle numbers with various solute concentrations for immiscible fluids at 0, 100, 1000 and 1 × 10^5^ time steps was presented in Fig. 4(a). The initial solute distribution was assigned following the same initialization procedure as that of in Fig. 2, i.e. the solute was randomly distributed around the average value of 0.45 with a range of slight fluctuation altitude of 0.05. In the time evolution, the range of solute variation becomes wider firstly and then two distinct peaks appear after 1000 steps. The two distinct peaks represent a solute-rich and another solute-poor liquid phases respectively. The solute compositions at the peaks are in agreement with the minimum Gibbs free energy positions indicated in Fig. 1, which is the cross point between the comment tangent line and the average chemical composition. It can be seen clearly from Fig. 4(a) that the two peaks at 10^5^ time steps are not in the same height. This is due to the initial average solute composition is 0.45 rather than 0.5 and the equilibrium solute composition positions is in symmetrical position regarding to 0.5 rather than 0.45. To prove that the level rule is valid in the method developed in the present work, another case with identical simulating conditions as that in Fig. 4(a) apart from the average composition value of 0.5 has been studied. The solute concentrations at 0, 100, 1000 and 1 × 10^5^ time steps was plotted in Fig. 4(b). The heights of two peaks at 1 × 10^5^ time steps are the same within fluctuation. The distribution of the particle numbers obeys the level rule in equilibrium phase diagram. The presence of two peaks in concentration distribution indicates the happening of solute demixing. This is in agreement with the thermodynamic prediction indicates in Fig. 1.

**Figure 5:**
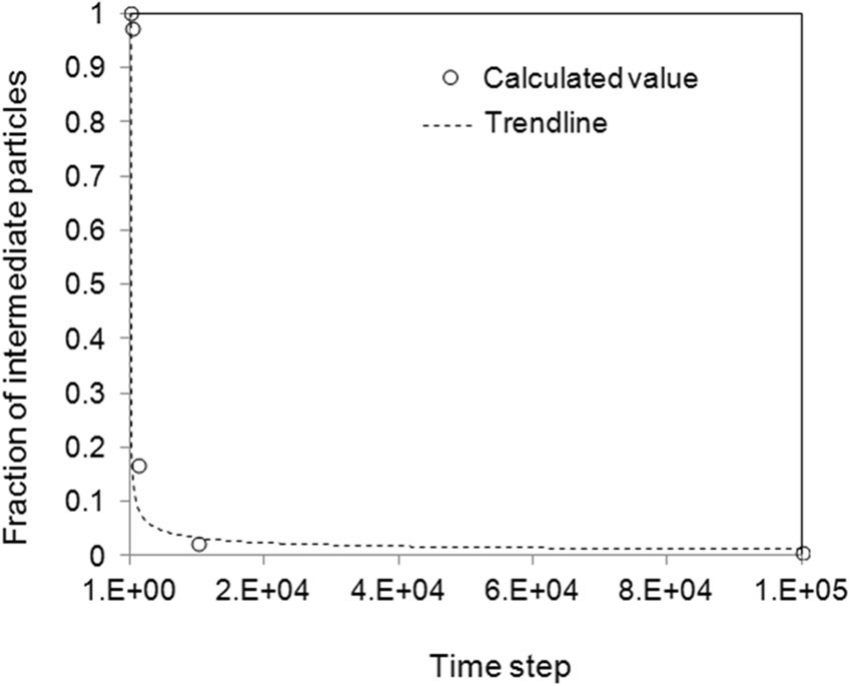
Evolution of the fraction of particles with intermediate solute composition. Trendline-fitting reveals scaling factor around −0.47.

**Figure 6:**
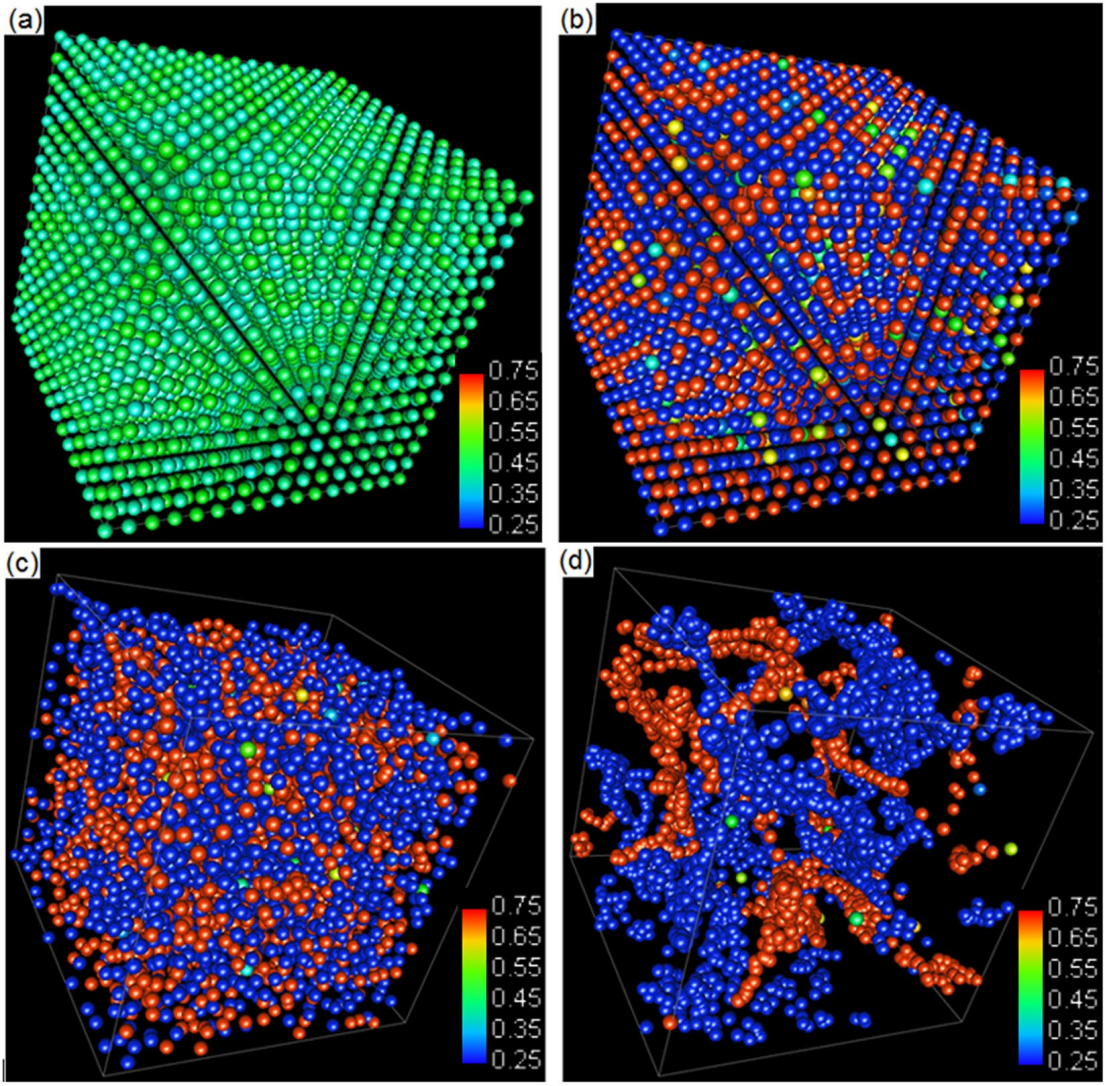
Particle solute distributions at 0, 10^3^, 10^4^, and 10^5^ time steps. The colour spectrums are from blue (0.25) to red (0.75).

The statistical calculation indicates some particles are with the solute composition between that of two equilibrium values, or called intermediate solute composition. The number of particles with intermediate solute concentration reduces drastically during the time evolution. Figure 5 presents the fraction of such particles at various time steps. In the beginning, the number of particles with intermediate solute composition reduces in a scaling law with a scale index of −0.47 according to the trend line fixing. It can be seen that that a large reduction of intermediate phase occurs at early stage and the reduction rate decreases drastically after the two distinct peaks appear. Some particles may be trapped at the interface between the solute-rich and solute-poor phases, and take longer time to evolve toward the peak area along with the phase coarsening and domain coalescence. At 10^5^ time steps the ratio of intermediate phase is less than 1%. The intermediate particle can be seen clearly in the 3D particle distribution figures shown in Fig. 6, where the solute compositions were indicated by particles’ colours from blue for 0.25 to red for 0.75. It can be seen clearly that intermediate particles are located in the interface area.

## Discussion

**Figure 7:**
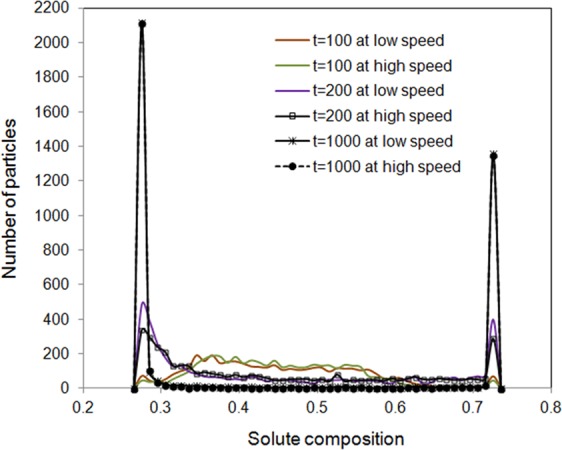
Effect of different initial random speed on the solute demixing at 100, 200 and 1000 time steps.

In order to understand how the initial speed of mesoscopic particles and substance diffusivity affect fluid kinetic behaviours, several pseudo simulations are performed for the immiscible fluids. For the low speed case, the initialization of particle speed components in 3 Cartesian coordinates were assigned randomly between −0.5 and 0.5 speed unit with average macroscopic speed of zero. In the high initial speed simulations, the variation was 1000 times larger as that of the low speed case. The solute configurations among particles at 100, 200 and 1000 time steps are presented in Fig. 7. Larger speed causes longer evolution time toward the equilibrium. This is understandable because larger fluctuation requests longer time to dissipate its kinetic energy^30^. However, it has been noticed that after 1000 time steps, the solute distributions in particles are not distinguishable for the two speed initialization cases. This is due to the definition of dissipative force in DPD method. Larger fluctuation of speed causes larger dissipative force, as is shown in ${\overrightarrow{F}}_{ij}^{D}=-\,\gamma {\omega }^{D}({r}_{ij})({\overrightarrow{r}}_{ij}\cdot {\overrightarrow{v}}_{ij}){\overrightarrow{r}}_{ij}$ for larger relative speed ${\overrightarrow{v}}_{ij}$. The initial speed condition will be smeared out. To keep the high speed, driving force will be required to the system, which is beyond the scope of the present work.

**Figure 8:**
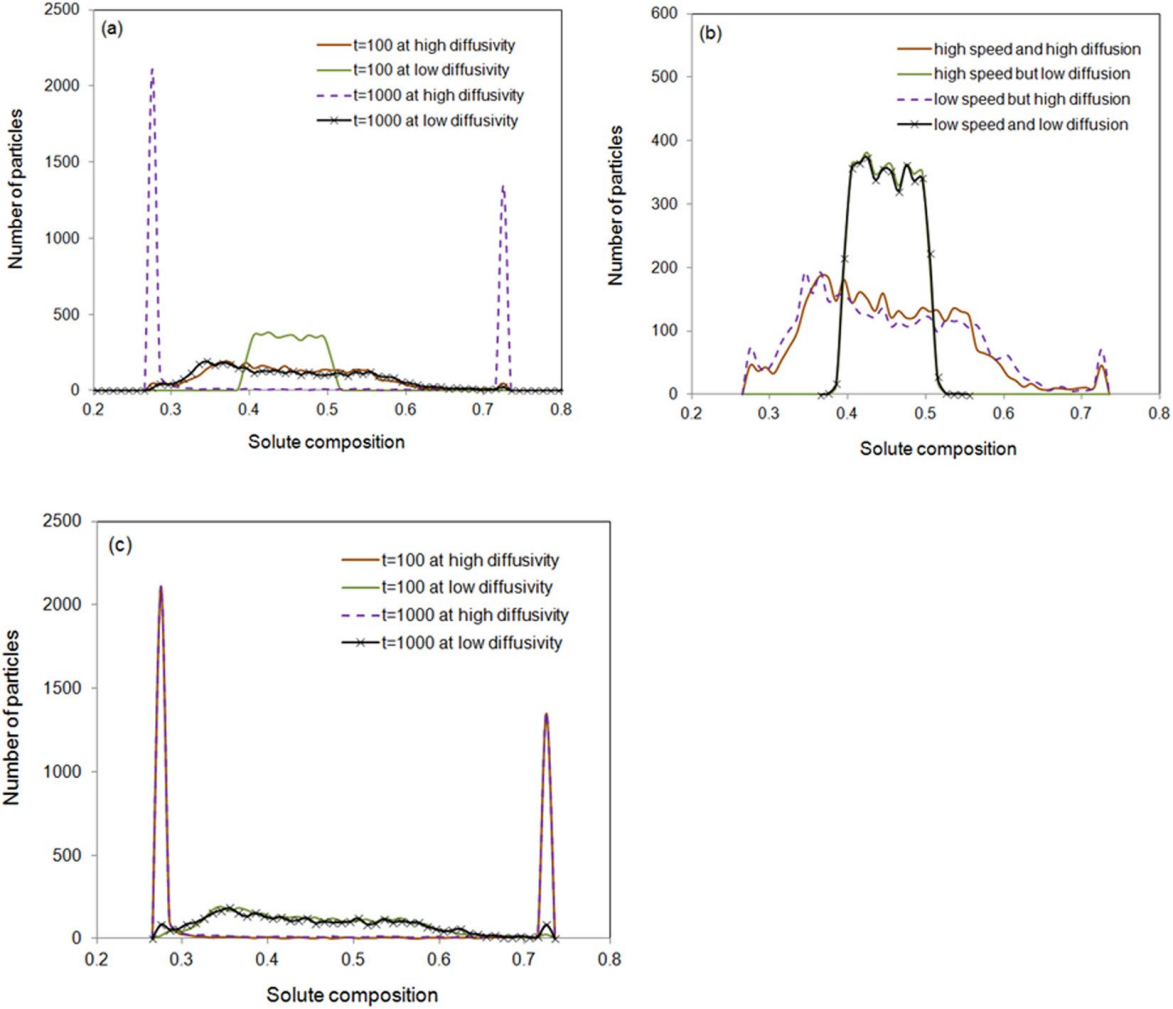
(**a**) Effect of diffusivity on solute demixing at 100 and 1000 steps. Comparison of the effects of initial speed and diffusivity on solute decomposition at (**b**) 100 and (**c**) 1000 time steps.

The effect of diffusivity on the system evolution has also been investigated. Figure 8(a) demonstrated the solute distribution for a low experimental diffusivity and another high speculate diffusivity (10 times of the true diffusivity) at 100 and 1000 time steps. It can be seen that diffusivity makes huge differences. Large diffusivity leads to a faster evolution toward thermodynamic equilibrium. Figure 8(b,c) demonstrate the comparison of effects of initial speed and diffusivity on the solute decomposition at 100 and 1000 time steps.

**Figure 9:**
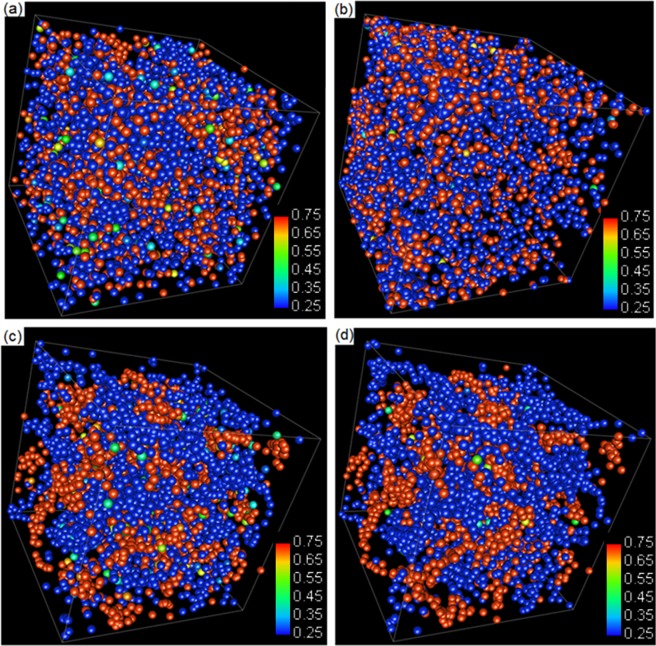
(**a**) High speed low diffusivity; (**b**) high speed high diffusivity; (**c**) low speed low diffusivity and (**d**) low speed high diffusivity. The spectrum is between 0.25 to 0.75.

Figure 9 plotted the particles solute distribution at 20000 time steps at four different conditions, namely the high speed with low diffusivity, high speed with high diffusivity, low speed with low diffusivity and low speed with high diffusivity. In the stage after many time steps of evolution, the microstructure evolution is not mainly due to diffusion but due to coalescence. The effect of diffusivity is not demonstrated clearly. Figure 9(a,b) are with high particle random speed and (c) and (d) with low speed. (a) and (c) have low diffusivity but (b) and (d) have high diffusivity. The high random speed helps to destroy the structures. This is similar to that of the effect of temperature on the fluid^31^.

In summary, a generic model that integrated the hydrodynamics, diffusion and thermodynamics has been developed. The method implemented the spirit of DPD and SPH and linked the theoretical frame into thermodynamics. Using the model, many engineering materials fabrication can be simulated. The parameters are determined by the real system. The results are in agreement with the phase equilibrium prediction made by computational thermodynamics. Their respective structural evolutions can also be shown in 3D space particle plots and some fine structure can be captured during the structure evolution. The method developed in the present work has some advantage and disadvantage aspects in comparison with other methods. It is better than lattice Boltzmann method in simulation of adiabatic condition and high Reynolds number fluids but worse than that in simulation of complex geometric boundaries^8,13^. It provides less accuracy than that of the phase field method in simulation of spinodal decomposition by diffusion but can deal with mixing and demixing in convection, in which phase-field method needs to integrate with another hydrodynamic method to achieve the task^10,32^. In comparison with conventional computational fluid dynamics, the method developed in the present work can provide information on microstructure evolution.

An understanding of how fluid speed and substance diffusivity affect phase separation/mixing as a case research has been given as well. The flow speed does not influence the concentration exchanges very much and then only have a subtle influence on the speed of phase separation/mixing while the substance diffusivity influences concentration exchanges a lot and have a big influence on the speed of phase separation/mixing. In terms of structural evolution, the substance diffusivity does not influence the structure evolution while the high speed flow violates forming fine structure at early stage and contributes to forming loose structure at late stage.

Several further works need to be done. An investigation of the influence of phase behaviours on fluid flows is still left out. A spatial temperature variation can be added to the model to accommodate temperature effects on fluid flow with phase behaviours. Moreover, as the framework of this model is generic, by replacing the thermodynamic law of phase behaviours with chemical reaction laws, a promising model for reactive fluids can be potentially developed, which will be very useful and meaningful in engineering applications.

## Methods

### Numerical algorithm

In combination of the modified velocity-Verlet numerical algorithm that was used in most DPD simulation^20^ and the leap-frog numerical algorithm that was applied in SPH simulation^33^, following numerical algorithm was implemented in the present work, where the iterations of velocity and solute concentration were divided into two half steps separated by the acceleration iteration.

14 $${r}_{i}(t+{\rm{\Delta }}t)={r}_{i}(t)+{\rm{\Delta }}t{v}_{i}(t)+\frac{1}{2}{({\rm{\Delta }}t)}^{2}{a}_{i}(t)$$

15 $${v}_{i}(t+\frac{1}{2}{\rm{\Delta }}t)={v}_{i}(t)+\frac{1}{2}{\rm{\Delta }}t{a}_{i}(t)$$

16 $${c}_{i}(t+\frac{1}{2}{\rm{\Delta }}t)={{\rm{c}}}_{i}(t-\frac{1}{2}{\rm{\Delta }}t)+{\rm{\Delta }}t\frac{d{c}_{i}(t)}{{\rm{dt}}}$$

17 $${a}_{i}(t+{\rm{\Delta }}t)={a}_{i}({r}_{i}(t+{\rm{\Delta }}t),{v}_{i}(t+\frac{1}{2}{\rm{\Delta }}t),{c}_{i}(t+\frac{1}{2}{\rm{\Delta }}t))$$

18 $${v}_{i}(t+{\rm{\Delta }}t)={v}_{i}(t)+\frac{1}{2}{\rm{\Delta }}t({f}_{i}(t)+{f}_{i}(t+{\rm{\Delta }}t))$$

19 $${c}_{i}(t+{\rm{\Delta }}t)={c}_{i}(t+\frac{1}{2}{\rm{\Delta }}t)+\frac{1}{2}{\rm{\Delta }}t\frac{d{c}_{i}(t)}{{\rm{dt}}}$$

where ${a}_{i}={{\rm{F}}}_{{\rm{i}}}/{{\rm{m}}}_{{\rm{i}}}=\sum _{{\rm{j}}}\,{{\rm{F}}}_{{\rm{ij}}}/{{\rm{m}}}_{{\rm{j}}}$ is the acceleration of particle *i* and Δ*t* is the time step.

### Software

The three-dimensional configurations of mesoscopic particles with various solute concentration were plotted using MatVisual software.

## Supplementary information

Supplementary Figure

## Data Availability

The datasets generated during and/or analysed during the current study are available from the corresponding author on reasonable request.
