# Supplementary material for: Mesoscale modelling of miscible and immiscible multicomponent fluids
Source: Sci Rep. 2019 Jun 4;9:8277. doi: 10.1038/s41598-019-44745-8 (PMC6547870; doi:10.1038/s41598-019-44745-8)
Supplement: Supplementary file 1 — Supplementary Figure [file 41598_2019_44745_MOESM1_ESM.docx]

# **Mesoscale modelling of miscible and immiscible multicomponent fluids**

Z. C. Zhao^1^, R. J. Moat^1^, R. S. Qin^1^*

1. School of Engineering & Innovation, The Open University, Walton Hall, Milton Keynes MK7 6AA, UK

* Corresponding author: [rongshan.qin@open.ac.uk](mailto:rongshan.qin@open.ac.uk)

^†^These authors contributed equally

Supplementary Figure


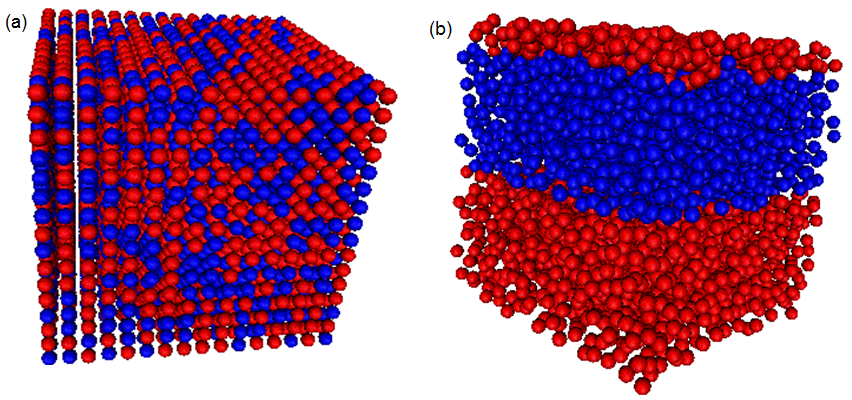


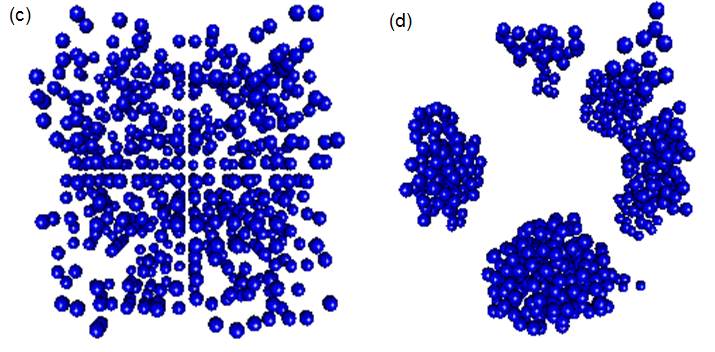


**Figure S1.** **The numerical results for particle demixing without diffusion** **using** $\boldsymbol{N}_{\boldsymbol{t}}\boldsymbol{=4000}$**,** $\boldsymbol{N}_{\boldsymbol{blue}}\boldsymbol{=1800}$ **for (a) t=0 and (b) t=9500 time steps; and** $\boldsymbol{N}_{\boldsymbol{blue}}\boldsymbol{=1000}$ **for (c)=0 and (d) t= 9500 time steps.** The blue particles are segregated into different geometries due to minimization of surface energy at periodic boundary conditions. When ${N_{blue}}/{N_{t}}<27\%$ the spherical particle has smallest interface area.
